# Electrostatic interaction of tumor-targeting adenoviruses with aminoclay acquires enhanced infectivity to tumor cells inside the bladder and has better cytotoxic activity

**DOI:** 10.1080/10717544.2017.1413450

**Published:** 2017-12-11

**Authors:** Soo-Yeon Kim, Whi-An Kwon, Seung-Pil Shin, Ho Kyung Seo, Soo-Jeong Lim, Yuh-Seog Jung, Hyo-Kyung Han, Kyung-Chae Jeong, Sang-Jin Lee

**Affiliations:** ^a^ Immunotherapeutics Branch, Research Institute, National Cancer Center Goyang Gyeonggi-do Korea; ^b^ School of Medicine, Institute of Wonkwang Medical Science, Wonkwang University, Wonkwang Univ. Sanbon Hospital Sanbon Korea; ^c^ Biomarker Branch, Research Institute, National Cancer Center, Center for Prostate Cancer, Hospital Goyang Gyeonggi-do Korea; ^d^ Department of Bioscience and Bioengineering, Sejong University Seoul Korea; ^e^ College of Pharmacy, Dongguk University-Seoul Goyang Gyeonggi-do Korea; ^f^ Translational Research Branch, Research Institute, National Cancer Center Goyang Gyeonggi-do Korea

**Keywords:** Aminoclay, bladder cancer, gene therapy, adenovirus, transduction

## Abstract

In a previous report, 3-aminopropyl functionalized magnesium phyllosilicate (aminoclay) improved adenovirus transduction efficiency by shielding the negative surface charges of adenovirus particles. The present study analyzed the physicochemical characterization of the electrostatic complex of adenoviruses with aminoclay and explored whether it could be utilized for enhancing tumor suppressive activity in the bladder. As a result of aminoclay-adenovirus nanobiohybridization, its transduction was enhanced in a dose-dependent manner, increasing transgene expression in bladder cancer cells and in *in vivo* animal models. Physicochemical studies demonstrated that positively charged aminoclay led to the neutralization of negative surface charges of adenoviruses, protection of adenoviruses from neutralizing antibodies and lowered transepithelial electrical resistance (TEER). As expected from the physicochemical properties, the aminoclay enabled tumor-targeting adenoviruses to be more potent in killing bladder cancer cells and suppressing tumor growth in orthotopic bladder tumors, suggesting that aminoclay would be an efficient, versatile and biocompatible delivery carrier for intravesical instillation of adenoviruses.

## Introduction

As the second most common cause of genitourinary cancer-related death (Siegel et al., 2013), urothelial carcinoma is the ninth most common cancer in the world. In particular, 70–80% of patients with urothelial carcinoma of the bladder are diagnosed with non-muscle invasive bladder cancer (NMIBC), and its standard treatment is transurethral resection (TUR) with or without intravesical therapy in order to decrease the risk of recurrence and progression to muscle invasive disease after TUR. Concerning intravesical chemotherapy, several strategies have been introduced to increase drug efficacy, such as electromotive drug administration (EMDA) and hyperthermia. However, quite a number of urothelial carcinoma patients experience recurrence after initial treatment, and then at least 30% among them progress to muscle-invasive disease in high-risk NMIBC. Therefore, more innovative and effective therapeutic strategies as the first line as well as second line have been needed for bladder cancer patients.

As a human pathogen, 57 serotypes of adenovirus have been identified and classified as A to G subgroups and have been known to be a causative agent for 5–10% of upper respiratory infections in children. Since the genetic materials of the adenoviruses are not incorporated into the host cell's genome post infection, these viruses are ideal for cancer gene therapy. The most commonly used adenovirus vector system is based on serotype 5 of species C. Serotype 5 primarily utilizes the coxsackievirus and adenovirus receptor (CAR) (Roelvink et al., 1998) for initial binding and then interacts with integrin molecules for internalization via clathrin-coated pits. Adenovirus-mediated gene therapy has been promising for treating various cancers and has also been investigated as a therapeutic approach for bladder cancer during previous decades. On top of safer dose-escalation inside the bladder, the intravesical instillation of the virus has demonstrated distinct advantages. First, the adenovirus comes in direct contact with the bladder cancer and, as a result, demonstrates potent anti-tumor activities (successful adenovirus-mediated wild-type p53 gene transfer) in patients with bladder cancer by intravesical vector instillation (Kuball et al., 2002; Benedict et al., 2004). Recent studies further demonstrated that adenovirus gene therapy can be a good combinatorial therapy with well-established standard therapies such as Bacillus Calmette-Guerin (BCG) or chemotherapy (Shieh et al., 2006; Li et al., 2017; Shore et al., 2017). Second, intravesical instillation of adenoviruses also eliminates the need for systemic administration through blood. Avoidance of the systemic circulation minimizes the immune system’s clearance of the virus and avoids viral elimination via liver tropism. However, efficient delivery and selective tumor-targeting still remain major hurdles to this type of treatment in the bladder.

With respect to efficient delivery, bladder cancer cells and tumors, especially those at an advanced state and of high grade, frequently lose CAR expression, which limits the use of adenoviruses to treat bladder cancer (Douglas et al., 2001). Numerous efforts have been attempted to overcome the limitations of adenoviral vector delivery posed by low CAR expression levels in many cell types. Since adenoviruses have negative surface charges and result in electrostatic repulsion from the cell membrane, those studies investigated the charge shielding with cationic vehicles and achieved significant improved infectivity (Connor et al., 2001; Yamashita et al., 2002; Kasman et al., 2009; Gosnell et al., 2014). We also reported cationic nanoparticles that shielded the anionic properties of the outer surfaces of the adenoviral particles and increased gene transfer in both CAR-positive and CAR-negative cells. Here, we further characterized the nanobiohybrid complex composed of aminoclay and adenoviruses, and evaluated whether it could be utilized for the development of bladder cancer therapy.

## Methods

### Cell lines and cell culture

Human bladder cancer cells (J82, T24, UMUC3) and mouse colon cancer cell CT26 cells were obtained from the American Type Culture Collection (Manassas, VA). The mouse bladder cancer cell MBT-2 was a kind gift from Dr. Kim WJ (Chungbuk National University, Korea) and mouse tonsil cancer cells (MEER and MLM) were kind gifts from Dr. Lee JH (Sanford Cancer Research Center, South Dakota, USA). UM-UC3 cells were maintained in MEM media supplemented with 10% fetal bovine serum and 1% penicillin/streptomycin. All other cells used in this study were maintained in RPMI 1640 media supplemented with 10% fetal bovine serum and 1% penicillin/streptomycin. Cells were always kept in a humid incubator at 5% CO_2_ and 37 °C.

### Aminoclay preparation and the complex formation of adenoviruses with aminoclay

The synthesis of aminoclay was described in a previous study (Kim et al., 2017). Briefly, 3-aminopropyltriethoxysilane of 11.7 mmol was added dropwise to magnesium chloride of 7.24 mmol prepared in 40 ml ethanol. A white precipitate formed and the resulting aminoclay was separated by centrifugation, washed with ethanol of 50 ml three times and dried in air at 40 °C. For exfoliation of the obtained aminoclay, the powder was dispersed in water and subjected to ultrasonication for 10 min. For complex formation of aminoclay with adenoviruses, adenoviruses and aminoclay were mildly mixed by gentle pipette tip aspiration, followed by incubation for 20 min at room temperature.

### Adenoviral gene transfer

A density of 1 × 10^5^ cells was prepared in six-well plates and the cells were kept in a CO_2_ incubator under 37 °C until 70–80% confluence. The culture media was replaced with 900 μl of serum free media and adenovirus-aminoclay complexes were added in a dropwise manner. After 4 hr incubation in a CO_2_ incubator at 37 °C, the cells were washed with phosphate-buffered saline (PBS) to remove the unbound complexes, and 2 ml of fresh culture media containing 10% serum were added. GFP expression was assessed after 24 hr additional incubation.

### RNA isolation and RT-PCR

Total RNA from each cell was isolated using a PureLink^TM^ RNA Mini Kit (Invitrogen, Carlsbad, CA). Synthesis of cDNA from the extracted RNA was performed for 1 hr at 37 °C using an Omniscript RT kit (QIAGEN, Valencia, CA). For the determination of the expression levels of mRNA, qPCR was conducted using DNA polymerase (Promega, Madison, WI). Primers used in PCR were in the following: 5′-CAG AAG CTA CAT CGG CAG TAA TCA-3′ and 5′-CTC TGA GGA GTG CGT TCA AAG TC-3’ for CAR, 5′-CAA GGT GAG CGG GAC CAT-3′ and 5′-TTG G\CA GAC AAT CTT CAA GCA-3′ for α_v_ Integrin, 5′-CCC TCG AAA ACC CCT GCT AT-3′ and 5′-TTA GCG TCA GCA CGT GTT TGT AG-3′ for β_3_ Integrin, 5′-GGC TGG GAC GTC ATT CAG AT-3′ and 5′-AGC TGG AAG GTG GTC TTG TCA-3′ for β_5_ Integrin, 5′-TGG TCA CCA GGG CTG CTT TTA -3′ and 5′-TCC TGG AAG ATC GTG ATG GGA-3′ for GAPDH, 5′-CCT GCT GAC CGT TCT TGG TA-3′ and 5′-CCT TCC CTG CCA CCT TGT AA-3′ for mouse CAR and 5′-GTC TTC CTG GGC AAG CAG TA-3′ and 5′-CTG GAC AGA AAC CCC ACT TC-3′ for mouse GAPDH.

### Measurement of trans-epithelial resistance (TEER)

MBT2 cells were seeded into Transwell^®^ Permeable Supports 24 mm Insert 6-well plates from Costar (Corning, NY) at a density of 1 × 10^5^ cells/wells. The epithelial barrier integrity of MBT2 was determined using an Epithelial Voltohmmeter EVOM^2^ and STX2 electrode from World Precision Instruments (Sarasota, FL). When TEER values were 600 ∼ 800 Ω·cm^2^, the grown cells were incubated with or without aminoclay for 4 hr in a serum-free condition. After 4 hr of incubation, the serum-free media condition was switched into a 2% serum condition and the transwell was incubated for 48 h in a CO_2_ incubator at 37 °C. TER value measurements were conducted at 4 hr, 8 h, 24 h, 48 h and 72 h.

### Zeta potential

For determining the electrical potential of complexes composed of adenoviruses and aminoclay, the zeta potential was measured using Zetasizer Nano ZS (Malvern, England). Adenovirus particles alone or adenovirus/aminoclay complexes (8 × 10^7^ PFU/500 μg) were diluted to 1 ml with deionized water from Sigma-Aldrich (St. Louis, MO). Default instrument settings and automatic analysis were used for all measurements.

### *In vitro* cytotoxicity assay

MBT2 cells were seeded into 96-well plates at a density of 5 × 10^3^ cells/well. When the confluence of cells reached almost 70–80%, cells were infected with varying concentrations of aminoclay with or without adenovirus in the absence of serum. After 24-h infection, cells were treated with 100 μM GCV (Roche, Cymevene Basel, Swizerland) in 100 μl of 2% serum media for 72 h. The cell viability was measured with an MTS assay (Promega, Madison, WI).

### *In vivo* studies in an orthotopic animal model

Five-week-old female C3H mice were purchased from Orient Bio (Seongnam, Korea) and kept for an acclimation period for 1 week. MBT2 cells expressing luciferase were instilled intravesically via the urethra using a catheter with 2 × 10^6^ cells in PBS. After 1 week, D-luciferin of 150 mg/kg was administered by intra-peritoneal injection and bioluminescence was detected by an IVIS Lumina XRMS In Vivo Imaging System (Perkin Elmer, Waltham, MA). The quantitative signal intensity was calculated and presented as regions of interest (ROIs). For the evaluation of tumor suppressive activity of adenoviruses in orthotopic animal models, bladder orthotopic tumors in the bladder were implanted as described above. Tumors were grouped by adenoviruses alone or adenovirus/aminoclay. Each group was infected intravesically for 2 h and then a GCV of 75 mg/kg was administered by intraperitoneal injection twice a day for 14 days. Growth of the tumor volume was determined by measuring bioluminescence every 3–4 days using IVIS.

### β-galactosidase assay

C3H mice harboring orthotopic tumors in the bladder were intravesically infected by Ad5CMVβgal (5 × 10^8^ PFU) or Ad5CMVβgal pre-complexed with aminoclay of 500 μg aminoclay for 2 hr. Ad5CMVβgal was a replication-deficient virus harboring the β-galactosidase (β-gal) gene under a CMV promoter. After infection, the bladder was emptied with PBS and mice were kept alive for the next 24 hr. On the next day, mice were euthanized by CO_2_ aspiration and bladders were harvested. The tissues were fixed in 4% paraformaldehyde for 24 h in a 4 °C refrigerator. After fixation, the tissues were incubated in β-gal staining buffer including N,N-dimethylformamide (SeraCare, Milford, MA) for 24 h in a CO_2_ incubator at 37 °C. Tissues stained with β-gal were embedded into paraffin and sectioned into 4 µm sections using a microtome. The sectioned tissues were observed using a digital micro-imaging device, DMD108 (Leica, Wetzlar, Germany).

## Results

### Downregulation of CAR expression in bladder cancer

**Figure 1.**
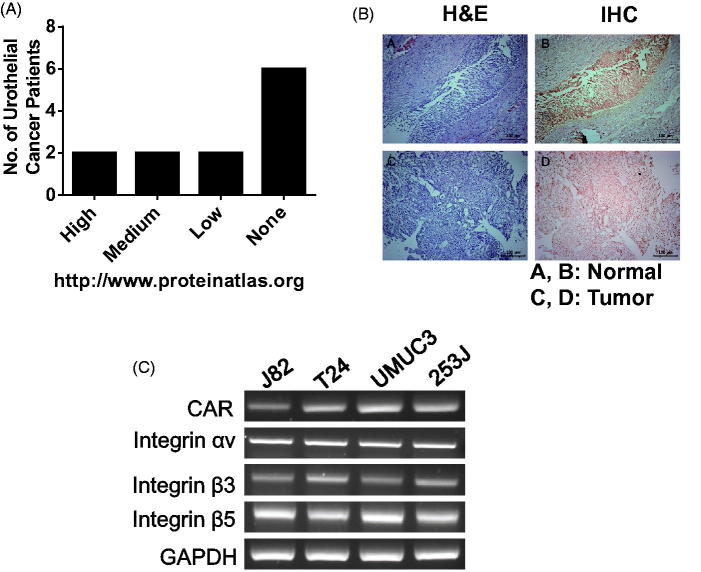
Downregulation of CAR proteins in bladder cancer. (A) CAR expression in bladder cancer according to a protein atlas (www.proteinatlas.org). (B) Bladder tumors and matched normal urothelium from NMIBC patients were harvested, fixed with 1% formaldehyde and were analyzed with anti-CAR antibodies following the standard protocol. (C) Bladder cancer cells were grown in 100 cm^2^ dishes and CXADR transcripts were evaluated using RT-PCR, as stated in the Methods.

CAR is a type-I membrane protein that is encoded by the CXADR gene and is utilized as a receptor by the group B coxsackie virus and subgroup C adenovirus. This protein is highly expressed in endothelial and epithelial cells. According to a public database (www.proteinatlas.org), CAR protein is highly prevalently stained with anti-CAR antibodies in a normal urinary bladder, and moderate to strong membranous staining was observed in 6 of 12 cancer patients. However, 6 bladder cancer patients had negative immunoreactivity with anti-CAR antibody as summarized in Figure 1(A). Tumors from 30% of NMIBC patients in this study exhibited the down-regulation of CAR proteins, as shown in Figure 1(B), in contrast to matched normal bladder tissue. Meanwhile, T24 and 253 J cells were determined to be low in CAR expression and were expected to be highly resistant to viral infectivity, but UMUC3 and J82 were much higher than those two types of cells (Figure 1(C)).

### Enhanced infectivity of adenoviruses to bladder cancer cells by aminoclay

**Figure 2.**
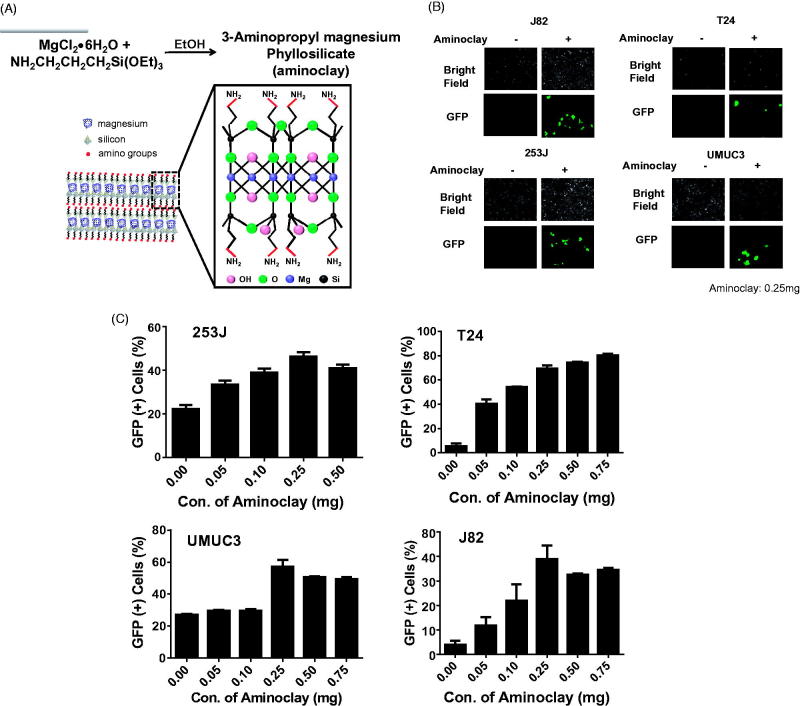
Enhancement of adenovirus infectivity to bladder cancer cells with the aid of aminoclay. (A) Aminoclay is synthesized in the presence of ethanol from magnesium silicate and has positive charges with multiple amino groups. (B) J82, T24, 253 J and UMUC3 were infected with different MOI of Ad5CMVGFP in the presence or absence of aminoclay: 100 MOI for J82, and T24, 10 MOI for 253 J and 5 MOI for UMUC3. (C) Cells were infected with a fixed dose of aminoclay of 250 μg plus Ad5CMVGFP and then observed under a fluorescence inverted microscope (20 × magnification). For counting GFP-expressing cells upon Ad5CMVGFP infection, Ad5CMVGFP was mixed with various concentrations, ranging from 0 to 750 μg, and incubated with 1 × 10^6^ cells for 24 h. GFP-expressing cells were determined by flow cytometry.

In the previous study (Kim et al., 2017), aminoclay complexation with adenoviruses changed the surface charge of the adenovirus particles and thus increased their binding to cellular membranes. Aminoclay was synthesized to have a branched amino group in ethanol and became positively charged, so that it could shield the surface negative charge of adenoviruses, as shown in Figure 2(A). To confirm that aminoclay could be a helper vehicle to facilitate the adenovirus entry to bladder cancer cells, GFP-expressing adenovirus was mixed with aminoclay of 0.25 mg prior to addition to each cell and was observed under fluorescence microscopy. Aminoclay was able to enhance the infectivity of adenovirus Ad5CMVGFP to all bladder cancer cells tested in a dose-dependent manner (Figure 2(B,C)).

### Effective gene delivery of adenoviruses with aminoclay

**Figure 3.**
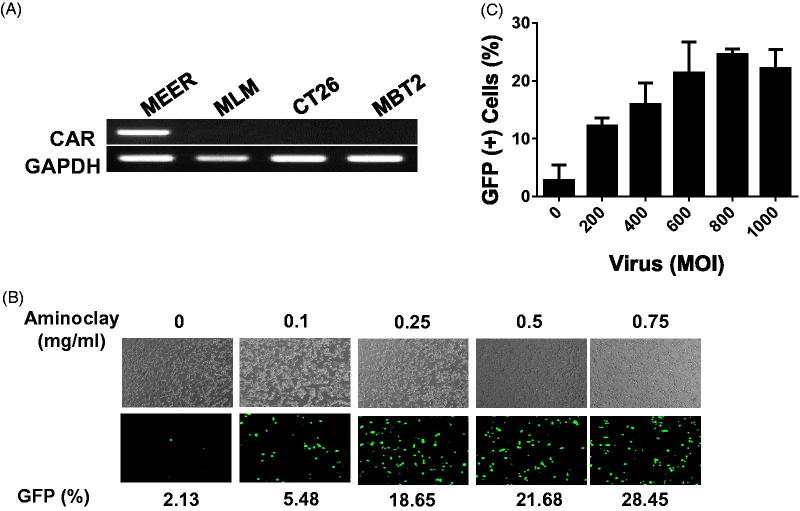
Enhanced adenoviral infectivity to MBT-2 aided by aminoclay. (A) Total RNA was obtained from cells prepared in 100-cm^2^ dishes and CAR expression was investigated by amplifying CAR transcripts in 1 µg of total RNA. (B) MBT-2 cells of 1 × 10^5^ were infected with Ad5CMVGFP of 8 × 10^7^ PFU alone or together with aminoclay of 0–1 mg aminoclay. GFP expression was observed under a fluorescence inverted microscope (20 × magnification). (C) The number of GFP-expressing MBT-2 cells was analyzed using flow cytometry at 24-h postinfection.

**Figure 4.**
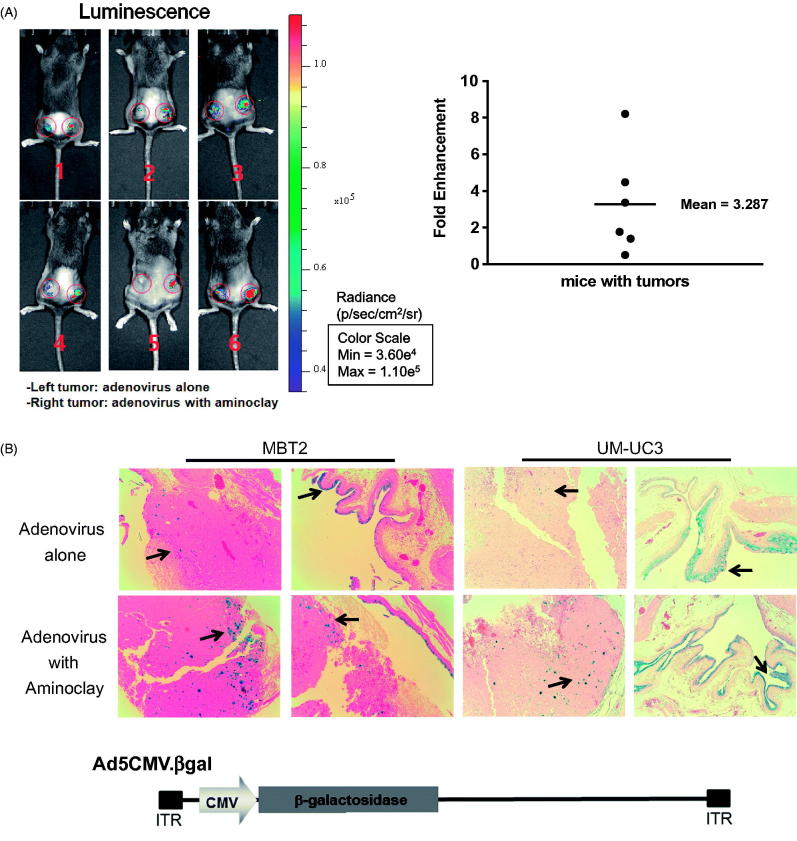
Enhanced infectivity of the adenovirus/aminoclay complex to a MBT-2 xenograft. (A) Subcutaneous MBT-2 tumors in C3H were implanted and were intratumorally injected with Ad5CMV luciferase of 5 × 10^8^ PFU alone or plus 500 μg of aminoclay. Bioluminescence was detected using the IVIS system (Perkin Elmer). ROI values of luminescence produced from luciferase gene expression and were presented as fold changes compared to adenovirus alone. (B) MBT-2/Luc bladder orthotopic tumors were implanted and tumor formations generating luminescence were confirmed, as described in the Methods. Tumors in the bladder were treated with Ad5CMVβgal of 5 × 10^8^ PFU alone or together with aminoclay of 500 μg. After 24 h, whole bladder organs were fixed with 1% formaldehyde and were sliced for β-gal assays. Ad5CMVβgal infectivity to tumors inside the bladder was compared under an inverted microscope (10× magnification).

The MBT-2 murine bladder cancer cell line was originated from a carcinogen-induced tumor of bladder epithelium in C3H mice. Implantation of MBT-2 cells in C3H mice is indispensable for the study of immunological aspects of therapies under development. As shown in Figure 3(A), CAR proteins in MBT-2, MLM and CT26 mouse cells are low in contrast to MEER, and they need a helper vehicle for efficient adenovirus transduction. In mouse tonsil cancer cells, MEER was included as a positive control. Therefore, we tested whether aminoclay could enhance the adenovirus transduction in MBT-2 as a helper vehicle. When mixed with aminoclay prior to addition to MBT-2 cells, the infectivity of Ad5CMVGFP to MBT-2 was increasing in a dose-dependent manner, as shown in Figure 3(B). Because 75 mg aminoclay exhibited cell damage (data not shown), all of the following experiments were done in the presence of 50 mg aminoclay. Next, we tested whether 50 mg of aminoclay could be sufficient for different numbers of adenovirus particles. Different numbers of Ad5CMVGFP adenovirus were mixed with 50 mg aminoclay, and then MBT-2 cells expressing GFP were counted with flow cytometry. As summarized in Figure 3(C), 50 mg aminoclay greatly increased the infectivity of all different numbers of adenoviral particles, confirming aminoclay to be the useful vehicle. To test whether aminoclay could enhance adenoviral transduction *in vivo*, we investigated whether aminoclay could enhance adenoviral infectivity in animal models. First, tumors were subcutaneously inoculated with MBT2 cells in C3H/He mice and then, were injected with adenovirus alone (left tumors) or with adenovirus as a complex of aminoclay (right tumors). As shown in Figure 4(A), tumors treated with the complex of adenovirus plus aminoclay showed the higher luminescence intensity. When we plotted the fold difference (Intensity_right_/Intensity_left_) (Figure 4(A), right panel), all six mice had increasing luminescence intensity in the case of adenoviruses with aminoclay. Next, we tested its enhancing activity in orthotopic bladder tumors of MBT2-Luc (Figure 4(B)) treated with Ad5CMVβgal adenoviruses. MBT2-Luc or UMUC3 cells were derived from MBT-2 or UMUC3 cells by transducing lentivirus expressing β-galactosidase and MBT2-Luc or UMUC3 tumors inside the bladder were detected by IVIS (Perkin Elmer, Waltham, MA, USA). Upon the luminescent intensity being captured, adenoviruses were instilled through the catheter and were permitted 2 h for infection. After 2 days, tumors from the bladder were harvested, fixed and then analyzed for β-galactosidase activity. As shown in Figure 4(B), aminoclay assisted with more effective infection to MBT2-Luc tumors, as expected from in vitro enhancement. Since β-gactosidase gene expression is controlled by the universal promoter CMV, both normal tissues and tumors were stained with β-gactosidase substrates. However, adenovirus mixed with aminoclay mediated the increased and stronger β-galactosidase gene expression. Such results suggest that aminoclay can augment the tumor suppressive activity, as long as the adenovirus has the selective tumor-targeting ability.

### Physicochemical properties appropriate for better infectivity of adenoviruses

**Figure 5.**
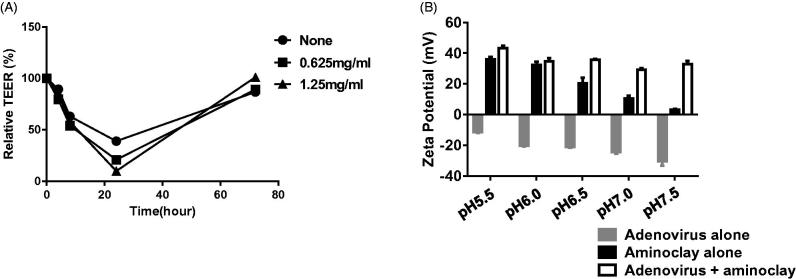
Physicochemical characteristics of adenoviruses complexed with aminoclay. (A) MBT-2 cells of 1 × 10^7^ were treated with PBS or 250 or 500 μg/ml of aminoclay. Membrane resistance was evaluated by measuring TEER. (B) Adenovirus of 8 × 10^7^ PFU and 500 μg of aminoclay were mixed and estimated for surface charges under different pH conditions.

Aminoclay requires an acidic environment and a low urine pH is associated with urothelial disorders and is an important risk factor for bladder cancer (Kadlubar et al., 1977). Urine is often acidic due to elevated levels of free aromatic amines and arylamine-DNA adducts originating from the bladder epithelium as demonstrated in several animal and human studies (Babu et al., 1996; Kadlubar et al., 1977, 1981, 1991) (Young & Kadlubar, 1982; Rothman et al., 1997). Assuming that urine from bladder cancer patients ranges between pH 5.5 and pH 7.0, aminoclay was evaluated to determine whether it could shield negatively charged adenoviruses at different pH values. Adenovirus was mixed with aminoclay and surface zeta-potentials were measured as described in Methods (Figure 5(A)). The zeta-potential of adenovirus alone was around −20 mV in pH 7.0 tris buffer with Mg^2+^, and aminoclay induced the surface charge of adenoviruses at approximately 0 mV regardless of pH changes, suggesting that aminoclay in acidic urine could effectively shield the negative charges of surface proteins, increasing the infectivity of adenoviruses to the bladder epithelium. Next, many pharmaceutical excipients have been known to disrupt the tight junctions of differentiated epithelial cells and enhance the bioavailability of adenoviruses *in vitro* and *in vivo* (Croyle et al., 2001). Therefore, the membrane integrity was tested upon addition of aminoclay to MBT-2 cells by measuring over a period of days (Figure 5(B)). When the adenovirus alone was added to the preparation of the MBT-2 cell layer, drops in TEER fell from 1000 to 900. The aminoclay formulation caused significant changes in TEER measurements, as it fell by 1100 ohms × cm^2^ for 48-h postapplication of the formulation. This study suggested that aminoclay enables the creation of bioavailable membranes for adenovirus transduction.

### Increased cytotoxicity of tumor-targeting adenoviruses

**Figure 6.**
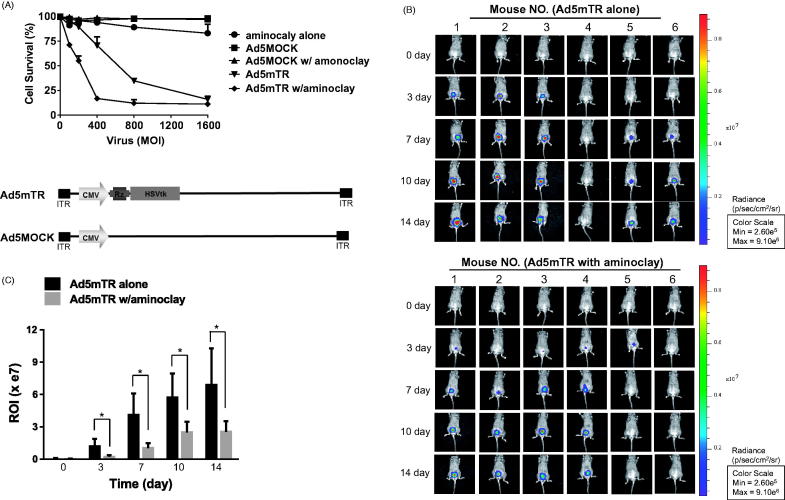
Enhanced tumor suppressive ability of adenoviruses in the presence of aminoclay. (A) MBT-2 cells of 1 × 10^4^ in 96-well plates were infected with increasing doses of Ad5MOCK or Ad5mTR. 500 μg of aminoclay were mixed with adenovirus prior to addition to cells. Cells were treated with prodrug GCV and their viabilities were measured 3 days later using an MTS kit. (B) MBT-2/Luc bladder orthotopic tumors were implanted and were instilled with tumor-targeting Ad5mTR of 5 × 10^8^ PFU alone or together with aminoclay. *In vivo* tumor suppressive activity by Ad5mTR was evaluated with ROI of luminescence taken by IVIS. (C) During in vivo tumor suppressive activity, ROI values were plotted at the different time points. * indicates the statistical significance. Two-tailed *p* values <0.05 were considered significant. STATA/SE version 10.1 software (StataCorp LP, College Station, TX) was used for the analysis.

Aminoclay-assisted cytotoxicity against MBT-2 cells was assessed, as shown in Figure 6. We measured the cytotoxic potential of the adenoviruses by incubating tumor cells with a range of virus particles for 7 days and then counting the live cells (Figure 6(A)). Here, we used adenovirus Ad5mTR-expressing herpes simplex virus thymidine kinase (HSV-TK) controlled by TERT-targeting trans-splicing ribozymes. HSV-TK is expressed in the presence of TERT mRNA (Goh et al., 2015). During these MTS assays, aminoclay enabled 250 MOI of adenovirus efficiently to kill 50% MBT-2 cells, but adenovirus alone required approximately 600 MOI to reach the same extent. Consistent with enhanced infectivity by the aid of aminoclay, aminoclay elicited more cytotoxicity by adenovirus Ad5mTR. Next, we investigated whether aminoclay could augment the tumor suppressive activity as adenoviruses were instilled into bladder through a catheter. MBT-2 cells expressing the luciferase gene were implanted inside the bladder and 1 × 10^9^ PFU of adenoviruses were injected with or without 50 mg of aminoclay. When the tumor-suppressive effects of the adenovirus/aminoclay complex were compared with adenoviruses alone, aminoclay was shown to help the anti-tumor activity of Ad5mTR with statistical significance with statistical significance (Figure 6(B,C)).

## Discussion

Bladder cancer is the second most common malignancy of the genitourinary tract. Urothelial carcinoma of the bladder (UCB) accounts for 80–90% of all bladder cancers. Approximately 75% of patients with UCB are initially diagnosed with NMIBC, which is found in either the mucosa or the submucosa (Kim et al., 2016). For patients with NMIBC, conservative management may allow the preservation of a functional bladder based upon TUR-BT, potentially combined with adjuvant intravesical therapy. However, 50–70% of patients experience disease recurrence within 5 years after TURBT and 10–20% of tumors progress to muscle invasion or higher-grade disease. Radical cystectomy with urinary diversion provides optimal control of progressive as well as recurrent NMIBC. Although advances in reconstructive techniques of the lower urinary tract, such as an orthotopic neobladder, have decreased the lifestyle changes associated with radical cystectomy, significant quality of life alterations occur after radical surgery. Adenovirus gene therapy has been developed as an alternative adjuvant therapy after TURBT, since intravesical BCG instillation or intravesical chemotherapy is due to ineffectiveness or side effects. Gene therapy poses several theoretical advantages over chemotherapy: (a) high selectivity for tumor cells with mutated genes, (b) restores cell growth to normalcy by correcting genetic defects rather than by killing cells, and (c) avoids the emergence of chemoresistance. Development of gene therapy in bladder cancer has focused on modifying the mutated urothelial cells and restoring normal functions of tumor suppressor genes. In previous studies, adenovirus-mediated gene therapies for bladder cancer patients have been developed in the following strategies: (1) to induce apoptosis by delivering tumor suppressor genes, such as p53 (Werthman et al., 1996); (2) to overexpress toxic genes (El-Zawahry et al., 2006); (3) to enhance host anticancer immunity by overexpressing immune modulators (Adam et al., 2007); and (4) to lyse cancer cells by virus amplification (Zhu et al., 2004). In those strategies, adenovirus-based gene therapy has demonstrated significant tumor-suppressive efficacy in preclinical models but has shown no significant benefits in the clinic. One of the reasons is adenovirus vector-mediated transduction is largely reduced by neutralizing antibodies reactive to adenoviruses (Roberts et al., 2006). When adenoviruses are directly injected into the tumor, transduction efficiency is also disturbed in the presence of high titers of neutralizing antibodies (Tomita et al., 2012). The other reason is that urothelium in the bladder becomes resistant to adenovirus infection due to poor CAR proteins and some tumor cells escape the adenovirus infection, leading to tumor relapses in human patients. Therefore, various strategies for enhancing adenovirus infectivity by bypassing have been introduced such as CAR-mediated endocytosis. Here, we explored whether the adenoviral infectivity to bladder cancer cells could be enhanced upon complexing with aminoclay. Aminoclay is less cytotoxic and biocompatible cationic materials can form electrostatic complexes with adenovirus particles, as reported in a previous study (Kim et al., 2017).

The bladder has two main barriers to being resistant to adenovirus transduction: (1) tight junctions in the urothelium, and (2) the mucopolysaccharide/glycosaminoglycan layer in the urothelium. A mucopolysaccharide/glycosaminoglycan layer in the urothelium obstructs efficient adenovirus transduction in orthotopic human tumor grafts and in rodent bladders (Watanabe et al., 2000). Therefore, several solutions have been suggested to overcome such a bona fide barrier by pre-treating urothelium with 30% ethanol (Engler et al., 1999) and polyamides, surfactants (e.g. dodecyl-beta-d-maltoside or sodium dodecyl sulfate) and Syn3 (a synthetic polyamide and low concentration of hydrochloric acid (60 mM) (Connor et al., 2001). Another barrier is the presence of tight junctions between the cells of an intact urothelium that is known to hinder adenoviral transduction (Chester et al., 2003). In this study, aminoclay seems to disturb tight junctions and expose more adenovirus receptors. It will be interesting to investigate the effect of treating urothelium and instilling adenovirus/aminoclay complexes for maximizing adenovirus transduction.

## Conclusions

Most adenovirus-mediated gene therapies have limiting effects on high-risk superficial bladder cancer patients in clinical trials. However, continuous investigations to improve the efficiency of adenovirus delivery, such as aminoclay, will contribute to its successful translation for cancer-suffering patients.
